# Intra- and inter-rater agreement between an ophthalmologist and mid-level ophthalmic personnel to diagnose retinal diseases based on fundus photographs at a primary eye center in Nepal: the Bhaktapur Retina Study

**DOI:** 10.1186/s12886-016-0295-0

**Published:** 2016-07-18

**Authors:** Raba Thapa, Sanyam Bajimaya, Renske Bouman, Govinda Paudyal, Shankar Khanal, Stevie Tan, Suman S. Thapa, Ger van Rens

**Affiliations:** Tilganga Institute of Ophthalmology, Kathmandu, Nepal; Vrije University Medical Center, Amsterdam, The Netherlands; Central Department of Statistics, Tribhuvan University, Kirtipur, Nepal; Vitreo-retina Service, Tilganga Institute of Ophthalmology, P O Box: 561, Kathmandu, Nepal

**Keywords:** Fundus photograph grading, Retinal pathology, Ophthalmologist, Mid-level ophthalmic personnel

## Abstract

**Background:**

Early detection can reduce irreversible blindness from retinal diseases. This study aims to assess the intra- and inter-rater agreement of retinal pathologies observed on fundus photographs between an ophthalmologist and two-mid level ophthalmic personnel (MLOPs).

**Method:**

A population-based, cross-sectional study was conducted among subjects 60 years and above in the Bhaktapur district of Nepal. Fundus photographs of 500 eyes of 500 subjects were assessed. The macula-centered 45-degree photographs were graded twice by one ophthalmologist and two MLOPs. Intra-rater and inter-rater agreements were assessed for the ophthalmologist and the MLOPs.

**Result:**

Mean age was 70.22 years ± 6.94 (SD). Retinal pathologies were observed in 55.6 % of photographs (age-related macular degeneration: 34.2 %; diabetic retinopathy: 4.2 %; retinal vein occlusion: 3.8 %). Twelve (2.4 %) fundus pictures were non-gradable. The intra-rater agreement for overall retinal pathologies, retinal hemorrhage, and maculopathy were substantial both for the ophthalmologist as well as for the MLOPs. There was moderate inter-rater agreement between the ophthalmologist and the first MLOP on second rating for overall retinal pathologies, [kappa *(k)*; *95 % CI* = 0.59 (0.51–0.66)], retinal hemorrhage [*k;* 95 *% CI* = 0.60 (0.41–0.78)], and maculopathy [*k;* 95 *% CI* = 0.52 (0.43–0.60)]. Inter-rater agreement between the ophthalmologist and the second MLOP for second rating was moderate for overall retinal pathologies [*k; 95 % CI* = 0.52 (0.44–0.60)], substantial agreement for retinal hemorrhage [*k; 95 % CI* = 0. 68 (0.52–0.84)], moderate agreement for maculopathy [*k; 95 % CI* = 0.59 (0.50–0.67)].

**Conclusion:**

There is moderate agreement between the MLOPs and the ophthalmologist in grading fundus photographs for retinal hemorrhages and maculopathy.

**Electronic supplementary material:**

The online version of this article (doi:10.1186/s12886-016-0295-0) contains supplementary material, which is available to authorized users.

## Background

Retinal disorders are among the leading cause of visual impairment and avoidable blindness, not only in developed but also in developing countries. Increases in life expectancy along with changes in lifestyle and dietary habits have resulted in increases in blindness from diabetic retinopathy (DR) and hypertension-related retinal problems in addition to age-related macular degeneration (AMD) [1–4]. Retinal disease has been identified as the major cause of blindness after cataracts in a population-based study conducted recently in Nepal [5, 6].

Several studies have shown strong agreement between ophthalmoscopy and fundus photograph grading for detecting DR as well as digital fundus imaging and stereoscopic fundus pictures for detecting AMD [7–11]. Similarly, there is good agreement between grading conventional 45° images and ultra-wide-angle images in detecting the macular pathology [12]. Therefore, digital imaging of the retina could be a useful tool for screening of retinal diseases in large epidemiological studies in both developed and developing counties.

The majority of retinal diseases are asymptomatic until there has been significant damage to the retina. Nepal has one ophthalmologist per 200,000 inhabitants and limited vitreo-retinal services, centered mainly in the urban areas. For this reason and lack of awareness, patients usually present late in the course of disease [13, 14]. Preservation of vision mainly depends on the timely detection and prompt treatment of sight threatening retinal conditions [15, 16]. Most districts in Nepal have district community eye centers which are primary level eye health facilities run by mid-level ophthalmic personnel (MLOP) who are trained to provide treatment for simple ocular problems. Training MLOPs to obtain and interpret fundus photographs depicting normal and abnormal retinal conditions could facilitate screening for visually threatening retinal diseases. This could lead to early diagnosis and timely referral of patients to an ophthalmologist.

This study will compare the agreement between MLOPs and an ophthalmologist regarding the grading of fundus photographs for the assessment of retinal pathologies. To the best of our knowledge, this is the first population-based study of its kind in Nepal. We hope, the findings from this study will be useful in setting up a system of screening of patients with retinal diseases by MLOPs, especially in remote areas of Nepal, and the results of this study could also be of importance and interest in developed countries.

## Methods

The Bhaktapur Retina Study is a population-based, cross-sectional study conducted to estimate the prevalence of vitreo retinal diseases among residents 60 years and older in the Bhaktapur district of Nepal. This is the second survey on the same cohort that was enrolled for the Bhaktapur Glaucoma Study conducted between 2007–2010. The study subjects were enrolled from August 2013 to December 2014 at Bhaktapur District Community Eye Center, a primary eye care facility. Detailed demographics of the study subjects have been described elsewhere [17]. Briefly, the second survey was conducted on the same population after a period of 5 years, primarily to study retinal disorders. Study participants were recruited from 30 clusters of the district, and a sample size of 2100 participants was calculated for the retinal study. All subjects attended the community eye centre in Bhaktapur district and underwent an ocular examination. A detailed history, biomicroscopic examination of the eye, including measurement of the intraocular pressure and a dilated fundus examination were conducted. Fundus photograph of the right eye of 500 cases were enrolled in the study for grading. The number of subjects calculated to reach 80 % power for assessing the intra-rater and inter-rater agreement was at least 475 [18]. The fundus picture was taken after dilating the pupil and comprised of macula-centered 45-degree single photographs using a Canon digital fundus camera (CRX, Canon Company). Trained ophthalmic photographers were involved in fundus photography.

A special questionnaire to rate retinal pathology was designed with reference to some studies conducted on diabetic retinopathy, age related macular degeneration, cataract [19–21] and also after consultation with ophthalmologists, public health experts and statisticians. The questionnaire was pre-tested by rating 50 fundus photographs. There was no problem while rating the photographs by an ophthalmologist and mid level ophthalmic personnel.

An ophthalmologist and two MLOPs independently graded the fundus photographs twice at three weekly intervals using a 17-inch computer monitor. No additional training was given between the first and second reading of fundus photographs. MLOPs are trained to deliver primary eye care at the district level. Before getting involved in the study, the MLOP received training regarding the grading of fundus photographs. The training conducted by the ophthalmologist was based upon diagnosing retinal diseases by interpreting several fundus photographs.

A questionnaire was developed in which the graders were asked to assess fundus photographs as normal or abnormal, presence or absence of retinal hemorrhages, and presence or absence of maculopathy and other retinal abnormalities. They were also asked to state when the fundus photo could not be graded. Details have been given in Additional file 1. DR was graded according to the Early Treatment Diabetic Retinopathy Study Criteria [15]. Likewise, AMD was categorized according to the International classification developed by the International ARM Epidemiological Study Group [22]. Both the intra-rater (the agreement between one grader) and inter-rater agreement (agreement between two different graders) were assessed in the study through the use of the kappa coefficient (*k*). The agreement was less than chance if kappa was <0.01, slight agreement if kappa was 0.01–0.20, fair agreement if kappa was 0.21–0.40, moderate agreement if kappa was 0.41–0.60, substantial agreement if kappa was 0.61–0.80 and almost perfect agreement if kappa was 0.81–0.99 [23]. The study was approved by the Institutional Review Board and Ethics Committee of Tilganga Institute of Ophthalmology (TIO) and conducted in accordance with the Declaration of Helsinki. Informed consent was written in the vernacular and was read to those unable to read. The subjects were asked to sign the consent form, and thumb impressions were taken for those unable to sign before enrollment into the study. Statistical analysis was performed using the STATA 9.0 (Stata Corp LD, College Station, Texas, USA).

## Results

Five hundred single fundus photographs of right eyes of 500 subjects were graded. The age ranged from 60 to 93 years with an average age of 70.22 ± 6.94 S.D. years. Females (54.8 %) outnumbered males. Retinal pathologies were observed in 55.6 % of study eyes on clinical examination by the ophthalmologist. AMD was the predominant retinal problem (34.2 %), followed by various grades of hypertensive retinopathy (4.6 %), diabetic retinopathy (4.2 %), retinal vein occlusion (3.8 %), and other retinal pathologies (6.6 %). Twelve (2.4 %) fundus pictures were not gradable due to media haze.

### Intra rater agreement of the ophthalmologist and MLOPs

There was substantial intra-rater agreement of the ophthalmologist for overall retinal pathologies [*k;* 95 % confidence interval (CI) = 0.72 (0.66–0.79)], retinal hemorrhage [*k;* 95 % CI = 0.74 (0.60–0.87)], and maculopathy [*k;* 95 % CI = 0.73 (0.65–0.80)]. The intra rater agreement of first MLOP was moderate for overall retinal pathologies [*k; 95 % CI* = 0.41 (0.34–0.49)] and retinal hemorrhage [*k; 95 % CI* = 0.51 (0.26–0.76)], and fair agreement for maculopathy [*k; 95 % CI* = 0.37 (0.28–0.45)]. Likewise, the intra-rater agreement of second MLOP was moderate for overall retinal pathologies [*k; 95 % CI* = 0.60 (0.53–0.68)], almost perfect agreement for retinal hemorrhage *k; 95 % CI =* 0.84 (0.72–0.97), and substantial agreement for maculopathy [*k; 95 % CI* = 0.70 (0.62–0.77)] (Table 1).

**Table 1:** Intra-rater agreement for fundus photograph reading by the ophthalmologist and by two MLOPs

Retinal findings	Ophthalmologist (Kappa value; 95 % CI)	MLOP 1 (Kappa value; 95 % CI)	MLOP 2 (Kappa value; 95 % CI)
Overall retinal pathologies	0.72 (0.66–0.79)	0.41 (0.34–0.49)	0.60 (0.53–0.68)
Retinal hemorrhage	0.74 (0.60–0.87)	0.51 (0.26–0.76)	0.84 (0.72–0.97)
Maculopathy	0.73 (0.65–0.80)	0.37 (0.28–0.45)	0.70 (0.62–0.77)
Non gradable	0.85 (0.77–0.92)	0.81 (0.73–0.89)	0.70 (0.59–0.81)

Abbreviations: *MLOP* mid-level ophthalmic personnel, *CI* confidence interval

### Inter rater agreement between ophthalmologist and MLOP on first rating

There was moderate inter-rater agreement between the ophthalmologist and the first MLOP for the first rating for overall retinal pathologies [*k; 95 % CI* = 0.44 (0.37–0.51)], retinal hemorrhage [*k; 95 % CI* = 0.41 (0.22–0.61)], and maculopathy [*k;* 95 *% CI* = 0.44 (0.34–0.55)]. Likewise, the inter-rater agreement between the ophthalmologist and the second MLOP for first rating was moderate for overall retinal pathologies [*k; 95 % CI* = 0.53 (0.45–0.61)], substantial agreement for retinal hemorrhage [*k;* 95 *% CI* = 0. 62 (0.46–0.78)], moderate agreement for maculopathy [*k; 95 % CI* = 0.58 (0.49–0.67)]. The inter-rater agreement between first MLOP and the second MLOP for first rating showed fair agreement for overall retinal pathologies [*k; 95 % CI* = 0.33 (0.25–0.41)], substantial agreement for retinal hemorrhage [*k; 95 % CI* = 0.63 (0.41–0.85)], and moderate agreement for maculopathy [*k*; *95 % CI* = 0.46 (0.36–0.56)] (Table 2).

**Table 2:** Inter-rater agreement between the ophthalmologist and two MLOPs at the first rating

Retinal findings	Ophthalmologist Vs MLOP 1 (Kappa value; 95 % CI)	Ophthalmologist Vs MLOP 2 (Kappa value; 95 % CI)	MLOP 1 Vs MLOP 2 (Kappa value; 95 % CI)
Overall retinal pathologies	0.44 (0.37–0.51)	0.53 (0.45–0.61)	0.33 (0.25–0.41)
Retinal hemorrhage	0.41 (0.22–0.61)	0.62 (0.46–0.78)	0.63 (0.41–0.85)
Maculopathy	0.44 (0.34–0.55)	0.58 (0.49–0.67)	0.46 (0.36–0.56)
Non gradable	0.75 0.66–0.84)	0.71 (0.61–0.81)	0.75 (0.65–0.84)

Abbreviations: *MLOP* mid-level ophthalmic personnel, *CI* confidence interval

There was moderate inter-rater agreement between the ophthalmologist and the first MLOP on second rating for overall retinal pathologies [*k*; *95 % CI* = 0.59 (0.51–0.66)], retinal hemorrhage [*k;* 95 *% CI* = 0.60 (0.41–0.78)], and maculopathy [*k;* 95 *% CI* = 0.52 (0.43–0.60)]. Likewise, the inter rater agreement between the ophthalmologist and the second MLOP for second rating was moderate for overall retinal pathologies [*k; 95 % CI* = 0.52 (0.44–0.60)], substantial agreement for retinal hemorrhage [*k; 95 % CI* = 0. 68 (0.52–0.84)], moderate agreement for maculopathy [*k; 95 % CI* = 0.59 (0.50–0.67)]. The inter-rater agreement between first MLOP and second MLOP for the second rating showed moderate agreement for overall retinal pathologies [*k; 95 % CI* = 0.58 [(0.51–0.66)], substantial agreement for retinal hemorrhage [*k 95 % CI* = 0.67 (0.49–0.84)], and maculopathy [*k; 95 % CI* = 0.65 (0.57–0.73)] (Table 3).

**Table 3:** Inter-rater agreement between the ophthalmologist and two MLOPs at the second rating

Retinal findings	Ophthalmologist Vs MLOP 1 (Kappa value; 95 % CI)	Ophthalmologist Vs MLOP 2 (Kappa value; 95 % CI)	MLOP 1 Vs MLOP 2 (Kappa value; 95 % CI)
Overall retinal pathologies	0.59 (0.51–0.66)	0.52 (0.44–0.60)	0.58 (0.51–0.66)
Retinal hemorrhage	0.60 (0.41–0.78)	0.68 (0.52–0.84)	0.67 (0.49–0.84)
Maculopathy	0.52 (0.43–0.60)	0.59 (0.50–0.67)	0.65 (0.57–0.73)
Non gradable	0.80 (0.72–0.88)	0.71 (0.62–0.81)	0.77 (0.68–0.86)

Abbreviations: *MLOP* mid-level ophthalmic personnel, *CI* confidence interval

There was substantial agreement on non-gradable fundus photographs for both intra- and inter-rater grading between the ophthalmologists and the MLOPs at both the first and second readings.

## Discussion

This is the first population-based study to compare agreement between an ophthalmologist and MLOPs grading fundus photographs for the assessment of retinal pathologies in Nepal. Visual impairment and blindness resulting from retinal diseases such as AMD, DR and retinal vein occlusion (RVO) are mainly due to the involvement of the macular area [13, 14, 24, 25]. AMD was the most common retinal condition among the study subjects, while DR and RVO were the next common retinal pathologies.

In our study, the intra-rater agreements for retinal hemorrhage and maculopathy were substantial both for the ophthalmologist as well as for one of the MLOPs.

This proves that proper training of MLOPs could be beneficial for screening of retinal diseases. There was moderate inter-rater agreement between the ophthalmologist and first MLOPs on the first rating and second rating of retinal hemorrhage and maculopathy. The inter-rater agreement between ophthalmologist and second MLOP for retinal hemorrhage was substantial in both of the ratings. The difference in rating agreement between the two MLOPs could be explained by individual levels of understanding of the retinal condition. The inter-rater agreement between the two MLOPs was also moderate for overall retinal pathologies and maculopathy at first rating except for retinal hemorrhage. For retinal hemorrhage, there was substantial inter-rater agreement between the MLOPs in the first as well as the second rating. Maculopathy had substantial agreement only in the second rating. Better agreement in the second rating was probably due to a better understanding of the retinal conditions by repeating the tests. Likewise, the obvious retinal lesions such as retinal hemorrhages were better identified while interpreting the fundus photographs.

Developed countries have adequate human resources and facilities for tele-ophthalmology, and testing of automated detection of AMD and DR screening of retinal disease is underway [26, 27]. The scenario is still very different in the developing world. In Nepal, there are only 175 ophthalmologists for a population of 28 million. The majority of the ophthalmologists are providing service in the urban areas, whereas more than two-thirds of the population resides in rural parts of the country. Even more so, tele ophthalmology is not a well-established model of screening for eye diseases. With the increase of diabetes and hypertension as a serious public health problem in Nepal, these models of screening need to be prioritized. AMD has been established as the most common retinal pathology and leading cause of blindness among elderly people in Nepal [5, 25]. Preservation of vision largely depends upon the early detection of such retinal conditions by using cost-effective screening methods conducted by MLOPs in countries with inadequate human resources.

There was exact agreement between ophthalmoscopy and fundus photo grading for detecting all grades of DR in studies conducted in the United States [7]. In another study conducted in the United States, a single field non mydriatric camera was superior to screen diabetic retinopathy over ophthalmoscopy [28]. Similarly, in another study, there was perfect agreement between digital fundus imaging and stereoscopic fundus pictures for detecting AMD which led the authors to conclude that digital imaging of the retina was useful for epidemiological studies [10]. Perfect agreement in detecting retinal hemorrhages by one of the MLOPs in our study proved that retinal diseases like DR and RVO may not have to be screened by an ophthalmologist. MLOPs providing services at the primary eye care level can screen retinal diseases and refer patients. This could help in reducing the irreversible blindness caused by retinal diseases.

Non mydriatric fundus cameras can be used for the screening of the majority of sight-threatening retinal conditions which affect the central part of the retina [29–32]; however, in our study, retinal examination was done after dilating the pupil in order not to miss any retinal pathology during clinical examination. Non mydriatic fundus photography is relatively easy and can be conducted to screen large number of patients in a short duration of time.

Due to the limited number of trained ophthalmologists, the government of Nepal has developed a course to certify MLOPs to provide primary eye care in ophthalmology. The curriculum for the MLOPs consists of a three-year training program after secondary school. It encompasses both general basic science and ophthalmology. They are also trained to carry out all related investigations in ophthalmology. Further enhancing their skill to interpret posterior segment eye diseases will help in screening of retinal conditions. The burden of diabetes and hypertension is expected to increase in the future, and by utilizing MLOPs in the screening of retinal diseases, this burden can be addressed. This will help in early detection of retinal diseases and prevention of blindness.

The moderate to fair agreement between one MLOP and the ophthalmologist warrants caution that some cases of retinal diseases could be missed. We hope that further training could improve the agreement between the two observers and can lead to a better rate of detection.

There are several strengths of the study. The large number of subjects and the setting where the study was conducted are major strengths. Primary eye care centres therefore can function as centres of screening in countries with limited resources. Comparing the results from two MLOPs was also the strength of the study.

In our study, elderly people with some media haze could have resulted in non-gradable pictures when the pupil had not been dilated for fundus photography. The use of a trained photographer and mydriatic photographs could have resulted in a larger number of gradable pictures.

## Conclusion

There is moderate to almost perfect agreement between MLOPs and ophthalmologist in grading fundus photographs for retinal hemorrhages and maculopathy. Utilization of MLOPs to screen retinal diseases at the primary eye care level can help in early detection and referral.

## Additional file

Additional file 1: Questionnaire of Intra- and inter-rater agreement between an ophthalmologist and mid-level ophthalmic personnel to diagnose retinal diseases based on fundus photographs at a primary eye center in Nepal: The Bhaktapur Retina Study. Title of data – Questionnaire of “Intra- and inter-rater agreement between an ophthalmologist and mid-level ophthalmic personnel to diagnose retinal diseases based on fundus photographs at a primary eye center in Nepal: The Bhaktapur Retina Study”. Description of data- structured questionnaire. (DOC 38 kb)
