# Supplementary material for: Intra- and inter-rater agreement between an ophthalmologist and mid-level ophthalmic personnel to diagnose retinal diseases based on fundus photographs at a primary eye center in Nepal: the Bhaktapur Retina Study
Source: BMC Ophthalmol. 2016 Jul 18;16:112. doi: 10.1186/s12886-016-0295-0 (PMC4950770; doi:10.1186/s12886-016-0295-0)
Supplement: Additional file 1: — Questionnaire of Intra- and inter-rater agreement between an ophthalmologist and mid-level ophthalmic personnel to diagnose retinal diseases based on fundus photographs at a primary eye center in Nepal: The Bhaktapur Retina Study. Title of data – Questionnaire of “Intra- and inter-rater agreement between an ophthalmologist and mid-level ophthalmic personnel to diagnose retinal diseases based on fundus photographs at a primary eye center in Nepal: The Bhaktapur Retina Study”. Description of data- structured questionnaire. (DOC 38 kb) [file 12886_2016_295_MOESM1_ESM.doc]

Nepal Eye Program

**Tilganga Institute of Ophthalmology**

Gaushala Kathmandu

**Research Department**

**Intra- and inter-rater agreement between an ophthalmologist and mid-level ophthalmic personnel to diagnose retinal diseases based on fundus photographs at a primary eye center in Nepal: The Bhaktapur Retina Study**

**Fundus Photo Grading**

**1.** Fundus Photo No **2.** Grader 1 Ophthalmologist

**3.** Grader Initials 2 Ophthalmic Assistant

**4.** Fundus Photo Grading 1 First grading

2 Second grading

**5.** Fundus Examination 1 Normal

2 Abnormal (If abnormal, go to question no. 6-9).

**6.** Maculopathy 1 Yes **7.** Retinal Haemorrhages 1 Yes

2 No 2 No

**8.** Non Gradable 1 Yes

2 No

**9.** Others specify ………………………………………..
